# Activation of Dll4/Notch Signaling and Hypoxia-Inducible Factor-1 Alpha Facilitates Lymphangiogenesis in Lacrimal Glands in Dry Eye

**DOI:** 10.1371/journal.pone.0147846

**Published:** 2016-02-01

**Authors:** Ji Hwan Min, Chul Hee Lee, Yong Woo Ji, Areum Yeo, Hyemi Noh, Insil Song, Eung Kweon Kim, Hyung Keun Lee

**Affiliations:** 1 Institute of Vision Research, Department of Ophthalmology, Yonsei University College of Medicine, Seoul, Korea; 2 Corneal Dystrophy Research Institute, Department of Ophthalmology, Yonsei University College of Medicine, Seoul, Korea; 3 Severance Institute for Vascular and Metabolic Research, Yonsei University College of Medicine, Seoul, Korea; National Institute of Dental and Craniofacial Research, UNITED STATES

## Abstract

**Purpose:**

By using hypoxia-inducible factor-1 alpha conditional knockout (HIF-1α CKO) mice and a dry eye (DE) mouse model, we aimed to determine the role played by delta-like ligand 4 (Dll4)/Notch signaling and HIF-1α in the lymphangiogenesis of lacrimal glands (LGs).

**Methods:**

C57BL/6 mice were housed in a controlled-environment chamber for DE induction. During DE induction, the expression level of Dll4/Notch signaling and lymphangiogenesis in LGs was measured by quantitative RT-PCR, immunoblot, and immunofluorescence staining. Next, lymphangiogenesis was measured after Dll4/Notch signal inhibition by anti-Dll4 antibody or γ-secretase inhibitor. Using HIF-1α CKO mice, the expression of Dll4/Notch signaling and lymphangiogenesis in LGs of DE-induced HIF-1α CKO mice were assessed. Additionally, the infiltration of CD45^+^ cells in LGs was assessed by immunohistochemical (IHC) staining and flow cytometry for each condition.

**Results:**

DE significantly upregulated Dll4/Notch and lymphangiogenesis in LGs. Inhibition of Dll4/Notch significantly suppressed lymphangiogenesis in LGs. Compared to wild-type (WT) mice, DE induced HIF-1α CKO mice showed markedly low levels of Dll4/Notch and lymphangiogenesis. Inhibition of lymphangiogenesis by Dll4/Notch suppression resulted in increased CD45^+^ cell infiltration in LGs. Likewise, CD45^+^ cells infiltrated more in the LGs of HIF-1α CKO DE mice than in non-DE HIF-1α CKO mice.

**Conclusions:**

Dll4/Notch signaling and HIF-1α are closely related to lymphangiogenesis in DE-induced LGs. Lymphangiogenesis stimulated by Dll4/Notch and HIF-1α may play a role in protecting LGs from DE-induced inflammation by aiding the clearance of immune cells from LGs.

## Introduction

Dry eye (DE) is a highly prevalent ocular inflammatory disorder affecting millions of people worldwide. However, disparities in the definition, diagnostic criteria, and treatment guidelines of the condition suggest that DE is a complicated heterogeneous disease involving many different pathophysiologic mechanisms.[1, 2] Although most DE patients complain of discomfort on the ocular surface area, the lacrimal gland (LG) is a major target organ of DE pathogenesis for both non-Sjögren DE and Sjögren syndrome.[3, 4] Inflammatory cytokines, inflammatory cells, and matrix proteases were upregulated after DE stress in human and mouse LGs. [5–7] Despite the importance of LGs and inflammation in DE pathophysiology, the exact mechanisms underlying increased inflammation in LGs affected by DE remain unknown.

According to previous studies, inflammatory conditions gave rise to new lymphatics extending into the cornea despite its immune privilege.[8, 9] Function-wise, lymphatics in cornea may facilitate the exit of antigen-presenting cells and antigenic material from the cornea to regional lymph nodes, thus promoting the induction of an adaptive immune response. [10] Similar to DE induced cornea, we found an increase of lymphatic vessels (LVs) in the LGs of a DE-induced mouse model.[11] By using immunofluorescence staining as well as immunoblot, upregulation of a well-known marker related to LV formation, lymphatic vessel endothelial hyaluronan receptor 1 (LYVE-1), was observed in LGs after DE stress.[11] Nonetheless, the underlying molecular mechanism for lymphatics growth in LGs and their functional role in the development of DE pathology have not yet been investigated.

Notch has been identified as an important factor for lymphangiogenesis interacting with hypoxia-inducible factor-1 alpha (HIF-1α).[12–14] Notch signaling performs diverse functions mediated by Notch receptors (Notch 1 –Notch 4), Delta-like ligands (Dll1, Dll3, Dll4), and Jagged ligands (Jagged 1 and Jagged 2).[15, 16] A previous research proved that the blocking Notch signaling pathway reduced LV sprouting during early postnatal development of wound healing in mouse dermis.[17] Moreover, conditional inhibition of Notch gene produced a disruption of normal ocular surface homeostasis, implying an important role played by Notch in the development of ocular surface disorders.[18]

The purpose of this study is to investigate DE-induced lymphangiogenesis in LGs, focusing on Dll4/Notch signaling and its relationship to HIF-1α activation by using mouse DE model.

## Methods

### Animal treatment and DE induction

Six- to eight-week-old male C57BL/6 mice (Charles River Laboratory, Wilmington, MA) were used in accordance with the standards set forth in the Association for Research in Vision and Ophthalmology Statement for the Use of Animals in Ophthalmic and Vision Research. The research protocol was approved by the Institutional Animal Care and Use Committee of the Yonsei University College of Medicine. DE was induced in the mice by placing them in a controlled-environment chamber (CEC). To achieve maximum ocular surface dryness, mice in the CEC (with a relative humidity below 13%) were given subcutaneous injections of 0.1 mL scopolamine hydrobromide (5 mg/mL; Sigma-Aldrich Chemical Co., St. Louis, MO) three times a day.

To suppress the activation of Dll4/Notch signaling, some groups of mice (n = 5 per group) were intraperitoneally injected with anti-Dll4 antibody (α-Dll4 Ab) (200 μg per day; Abcam^®^, Inc., Cambridge, MA) or γ-secretase inhibitor (GSI) (5 mg/kg per day; Sigma-Aldrich Chemical Co.) daily. By inhibiting the cleavage of Notch intracellular domain from its activated receptor, GSI has been proven to be effective in downregulation of Notch signaling.[19] As a control of the α-Dll4 Ab group, hamster IgG was given (n = 5; 200 μg per day; Abcam^®^, Inc.). As a negative control, DMSO was administered (n = 5; 5 mg/kg per day; Sigma-Aldrich Chemical Co.).

To generate HIF-1α conditional knockout (CKO), mouse mammary tumor virus (MMTV)-Cre mice (The Jackson Laboratory, Bar Harbor, ME) and HIF-1α floxed mice (The Jackson Laboratory) were used. The viral MMTV promoter directed the expression of Cre recombinase in the secretory epithelium of mammary glands, salivary glands, and LGs. The down regulation of HIF-1α in LGs was confirmed by immunoblot and IHC staining. The detailed methods for generating HIF-1α CKO have been described in our earlier work.[11]

### Tissue preparation

After 10 days of DE induction, the mice were sacrificed and LGs were collected. Each tissue was halved and was either fixed in 3.7% paraformaldehyde for immunofluorescence staining or stored at -70°C for quantitative Real-Time-PCR (qRT-PCR) and immunoblotting.

### Tissue RNA extraction and qRT-PCR

RNA was isolated using an RNeasy Micro Kit (QIAGEN, Valencia, CA) from mouse LGs, and reverse transcription was performed using a Superscript III Kit (Invitrogen, Carlsbad, CA). Real-time qPCR was performed using SYBR® Premix Ex Taq (Takara Bio Inc., Otsu, Japan) with a StepOnePlus Real-Time PCR System (Applied Biosystems, Foster City, CA). Preformulated primers were used in order to evaluate mRNA expression. Detailed primer information is described in Fig A in S1 Fig.

### Immunohistochemical staining and immunofluorescence staining

LGs were harvested and analyzed by immunohistochemical (IHC) staining and immunofluorescence staining. Histologic sections (5 to 7 μm) were collected on poly-L-lysine-coated slides and deparaffinized. The sections were then rehydrated with a xylene-grade alcohol scale and rinsed with phosphate-buffered saline. Sections were blocked with rabbit/goat/rat serum for 40 minutes at room temperature and exposed to primary antibodies: NOTCH1 (Goat polyclonal anti-mouse, 2μg/ml; Santa Cruz Biotechnology, Inc., Dallas, TX), DLL4 (Rabbit polyclonal anti-mouse, 1μg/ml; Abcam^®^, Inc.), LYVE-1 (Rat monoclonal anti-mouse, 2μg/ml; Santa Cruz Biotechnology, Inc.). Antibodies were diluted from 1:100 to 1:200 and incubated overnight at 4°C. After washing in Tris-buffered saline supplemented with Tween 20 (TBST), each section was exposed to secondary antibodies for 1 hour. After washing out the secondary antibodies with TBST, the sections were exposed to 4',6-diamidino-2-phenylindole (DAPI) (PureBlu™, Bio-Rad, Inc., Hercules, CA). The IHC staining method for LG has been described previously.[11] Anti-CD45 antibody (Rabbit polyclonal anti-mouse, 0.5 μg/ml; Abcam^®^, Inc.) was used for IHC staining of inflammatory cells. Light microscopy (Axio Imager 2, Carl Zeiss, Germany) was used for examination.

### Immunoblotting with LG sample

Total protein concentrations of supernatant fractions were determined using the bicinchoninic acid (BCA) protein assay (Bio-Rad, Inc.). Equal amounts of protein aliquots were boiled in equal volumes of 2× SDS Laemmli sample buffer and resolved on 8% or 10% (w/v) with primary antibodies; anti-NOTCH1 (0.2μg/ml; Abcam^®^, Inc.), anti-Dll4 (0.1μg/ml; Abcam^®^, Inc.), anti-LYVE-1 (1μg/ml; Abcam^®^, Inc.) and anti-PECAM (2μg/ml; Abcam^®^, Inc.). Immunoreactive bands were detected with horseradish peroxidase-conjugated secondary antibodies and visualized using the enhanced chemiluminescence technique.

### Statistical analysis

The statistical analysis of more than three groups was performed by One-way ANOVA. As post hoc analyses, the subgroups were analyzed by Newman-Keuls method, a stepwise multiple comparisons procedure used to identify sample means that are significantly different from each other. Additionally, Student t-test was performed to compare the two group samples. Dunnett’s test was also used to compare each treated group to the control group. A p-value of < 0.05 was considered to indicate a significant difference.

## Results

### DE induction upregulates Dll4/Notch signaling in LGs

**Fig 1:**
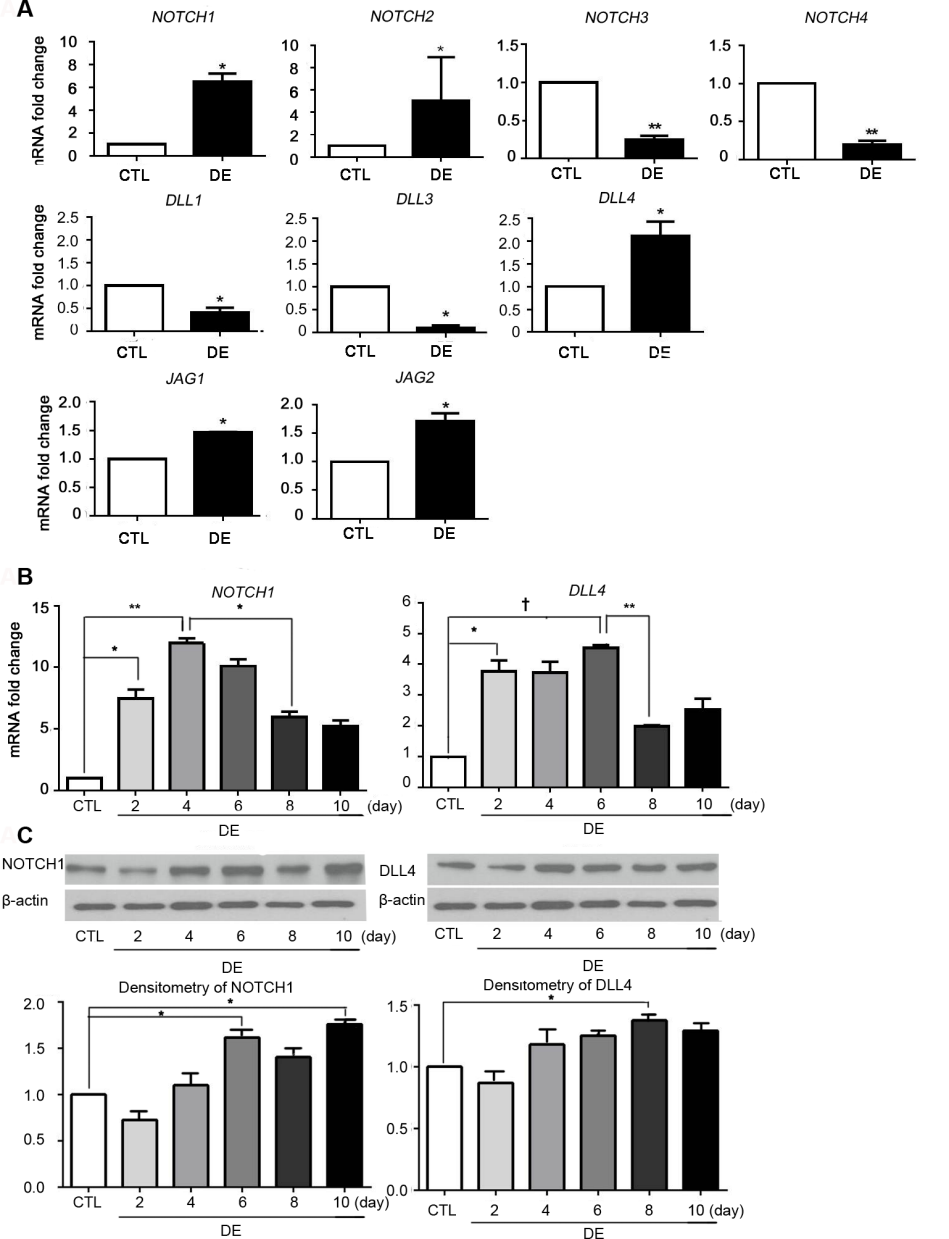
Change in NOTCH signaling in dry eye-induced lacrimal glands. After C57BL/6 mice were housed in a CEC with scopolamine administration for 10 days, LGs were obtained and prepared for qRT-PCR, and (A) mRNA levels of NOTCHs, DLLs, and JAGs were measured. (B) The change in the mRNA level of NOTCH1/DLL4 during DE induction was measured (C) During DE induction, each LG samples were prepared for immunoblot for NOTCH1 and DLL4 at Day 2, Day 4, Day 6, Day 8, and Day 10. Densitometry for protein concentreation quantification was done by using ImageJ software. Student’s t-test for statistical analysis: *p<0.05, **p<0.01, †p<0.001. Error bars indicate standard deviation. (CTL = normal control; DE = dry eye).

The expression level of Notch signaling was evaluated during DE induction. The qPCR results showed a significant increase of NOTCH1 and NOTCH2 expression at Day 10 (NOTCH1: mean 6.5 fold change, p = 0.034; NOTCH2: mean 5.1 fold change, p = 0.045). The mRNA level of Dll4, Jagged (JAG) 1 and JAG2 increased with significance at Day 10 (Dll4: mean 2.2 fold change, p = 0.032; JAG1: mean 1.5 fold change, p = 0.043; JAG2: mean 1.69 fold change, p = 0.041). The mRNA level of NOTCH1 and Dll4 at Days 2, 4, 6, 8, and 10 was measured. NOTCH1 and Dll4 expression started to rise at Day 2 (NOTCH1: p = 0.022; Dll4: p = 0.034). NOTCH1 reached the peak at Day 4 and declined significantly at Day 8. For Dll4, the mRNA level peaked at Day 6, with significant downregulation starting on Day 8 (Fig 1B). Immunoblot and densitometry of NOTCH1 showed peaks at Day 6 and Day 10, while Dll4 showed the highest level at Day 8 (Fig 1C).

### DE induction upregulates lymphangiogenesis in LGs

**Fig 2:**
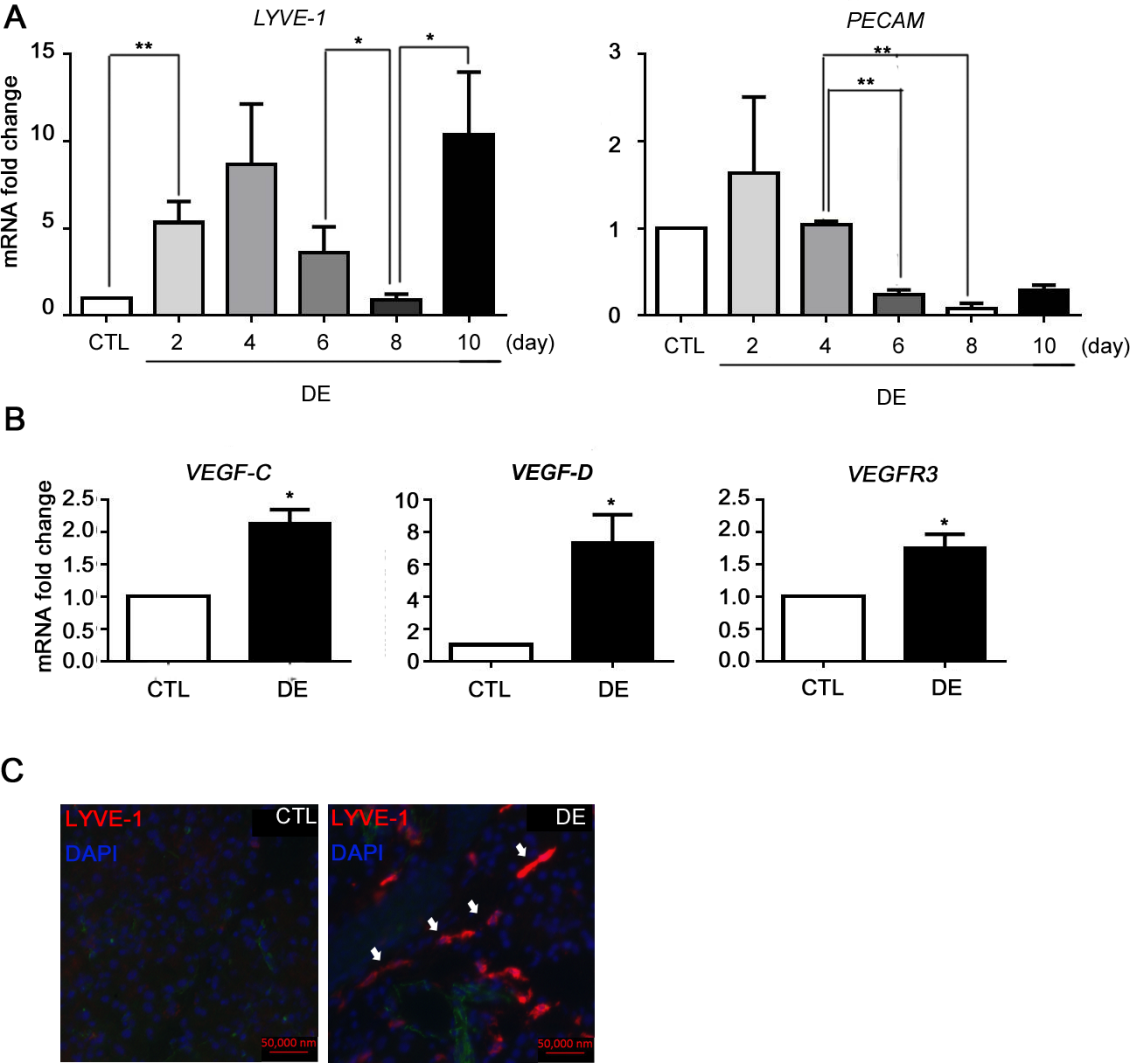
Change in LYVE-1, PECAM, VEGF-C, VEGF-D, and VEGFR3 expression in dry eye-induced lacrimal glands. During 10 days of DE induction, LGs were obtained and prepared for qRT-PCR and (A) the mRNA level of LYVE-1 and PECAM was measuredat Day 2, Day 4, Day 6, Day 8, and Day 10 of DE induction. (B) The mRNA levels of VEGF-C, VEGF-D, and VEGFR3 were measured at Day 10. (C) Immunofluorescence staining of LYVE-1 for DE and control group was performed at Day 10. Student’s t-test for statistical analysis: *p<0.05, **p<0.01. Error bars indicate standard deviation. (CTL = normal control; DE = dry eye).

To detect the level of LV and BV formation in LG in DE, the mRNA level of LYVE-1, PECAM, VEGF-C, VEGF-D, and VEGFR3 was measured. LYVE-1 is a powerful indicator for LVs, and PECAM is a representative marker for angiogenesis. These markers were used to assess the effects of DE stress on LV development and angiogenesis. Interestingly, the mRNA level of LYVE-1 showed biphasic humps, peaking twice at Day 4 and Day 10. A significant drop from Day 6 to Day 8 was observed (p = 0.028). From Day 8 to Day 10, LYVE-1 increased significantly with a mean 11.2 fold change (p = 0.045). After the mRNA level of PECAM showed an increase at Day 2 of DE induction (p = 0.245), there was significant downregulation of PECAM from Day 4 to Day 6 (p = 0.008), reaching the lowest level at Day 8 (Fig 2A).

The change of expression of VEGF-C, VEGF-D, and VEGFR3 was evaluated at Day 10 by qPCR. All three were upregulated with a mean 2.1 fold increase for VEGF-C (p = 0.036), a 7.3 fold increase for VEGF-D (p = 0.029), and a mean 1.8 fold increase for VEGFR3 (p = 0.047) (Fig 2B). Conclusively, immunofluorescence staining demonstrated an increased expression of LYVE-1^+^ cells at Day 10 (Fig 2C).

### Inhibition of Dll4/Notch signaling reduces lymphangiogenesis in DE-induced LGs

**Fig 3:**
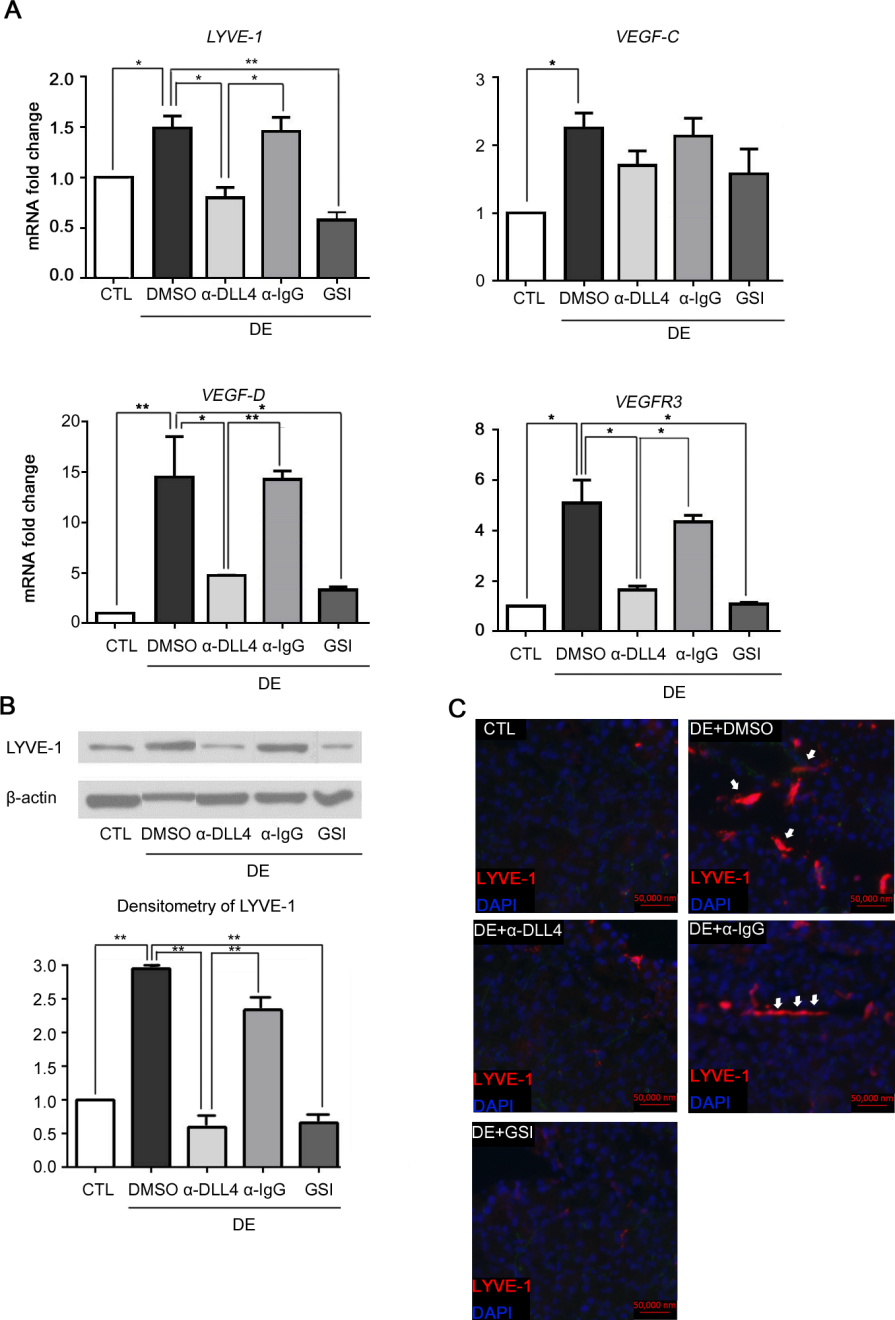
Change in LYVE-1, VEGF-C, VEGF-D, and VEGFR3 expression after inhibition of the NOTCH1-Dll4 axis by intraperitoneal injection of monoclonal anti-Dll4 antibody and γ-secretase inhibitor. While C57BL/6 mice were housed in a CEC with scopolamine administration for 10 days, several groups of mice were administered anti-Dll4 Ab and GSI for inhibiting the NOTCH1-DLL4 axis. Anti-IgG Ab was administered as a control for the anti-Dll4 antibody group, and DMSO was administered as a negative control. LGs were obtained after 10 days of DE induction and were prepared for qRT-PCR, immunoblot, and immunostaining. (A) The mRNA levels of LYVE-1, VEGF-C, VEGF-D, and VEGFR3 were measured using qPCR for each group. (B) Immunoblot and densitometry of LYVE-1 were measured for each group. (C) Immunofluorescence staining of LYVE-1 was performed for each group. Student’s t-test for statistical analysis: *p<0.05, **p<0.01. Error bars indicate standard deviation. (CTL = normal control; DE = dry eye; α-Dll4 = anti-Dll4 antibody; α-IgG = anti-IgG antibody; GSI = γ-secretase inhibitor; DMSO = dissolved dimethyl sulfoxide).

To investigate the role played by Dll4/Notch signaling in LV formation of DE-induced LGs, an inhibition study was performed. At Day 4, GSI and anti-Dll4 antibody did not manifest inhibitory effect on the mRNA level of LYVE-1 (Fig A in S2 Fig). However, the qPCR data at Day 10 showed a significant decline in LYVE-1 expression as compared to the vehicle (DMSO) group as shown in Fig 3A (GSI: p = 0.006, anti-Dll4 antibody: p = 0.033). Furthermore, the mRNA level of VEGF-D and VEGFR3 in the GSI and anti-Dll4 antibody group showed a significant reduction as compared to the DMSO group at Day 10 (for VEGF-D, GSI: p = 0.030, anti-Dll4 antibody: p = 0.039; for VEGFR3, GSI: p = 0.021, anti-Dll4 antibody: p = 0.040). However, although the VEGF-C was slightly downregulated, this change was not statistically significant (GSI: p = 0.191, anti-Dll4 antibody: p = 0.176 compared to DMSO). Anti-Dll4 antibody injection also effectively suppressed the mRNA levels of LYVE-1, VEGF-D, and VEGFR3 compared to those of its control anti-IgG antibody injected group (Fig 3A, LYVE-1: p = 0.037, VEGF-D: p = 0.009, VEGFR3: p = 0.041). The suppression of LYVE-1 by GSI and anti-Dll4 antibody was confirmed by immunoblot and immunofluorescence staining (Fig 3B and 3C).

### Downregulation of Dll4/Notch signaling and lymphangiogenesis in DE-induced LG of HIF-1α CKO mice

HIF-1α has been regarded as an important regulator for lymphangiogenesis in mouse models.[14] Additionally, we have previously discovered that HIF-1α CKO mice exhibit reduced expression of lymphatics.[11] Therefore, a study for demonstrating the interaction between HIF-1α, Dll4/Notch 1 signaling, and lymphangiogenesis in DE LGs was performed.

**Fig 4:**
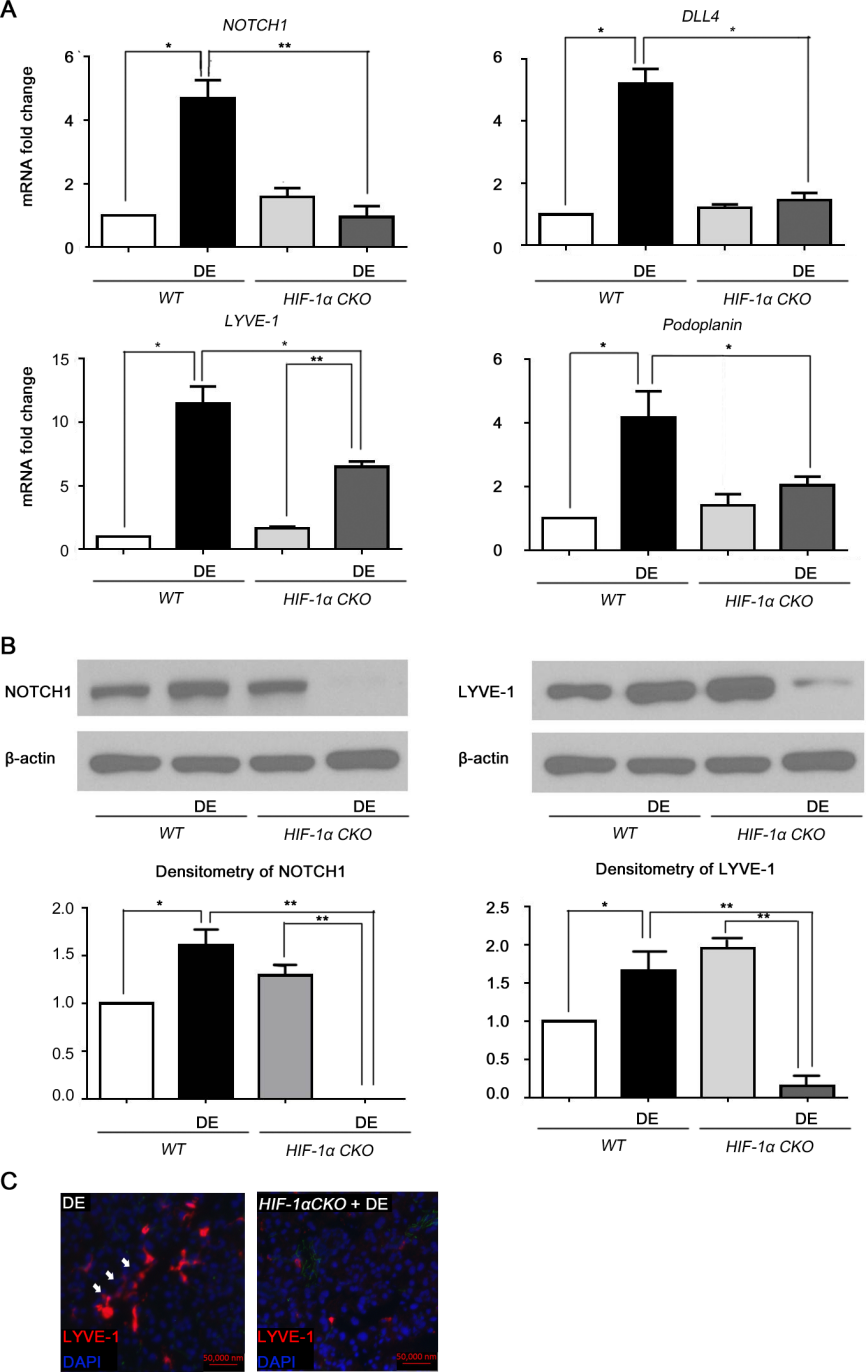
Change in NOTCH signaling and LYVE-1 expression in dry eye-induced HIF-1α conditional knockout mice. WT B6 and HIF-1α CKO mice were housed in a CEC with scopolamine administration for 10 days. LGs were obtained and prepared for qRT-PCR and (A) The mRNA level of NOTCH1, DLL4, LYVE-1, and Podoplanin at Day 10 was measured. (B) Immunoblot and densitometry for NOTCH1 and LYVE-1 were measured at Day 10. (C) Immunofluorescence staining of LYVE-1 was performed for DE-induced WT B6 mice and DE-induced HIF-1α CKO mice at Day 10. Student’s t-test for statistical analysis: *p<0.05, **p<0.01. Error bars indicate standard deviation. (WT = wild-type; DE = dry eye; HIF-1α CKO = HIF-1α conditional knockout).

The mRNA level of NOTCH1, Dll4, Podoplanin, and LYVE-1 were studied for DE-induced HIF-1α CKO mice. Podoplanin was measured in addition to clarify whether the change of LYVE-1 truly represents LVs because LYVE-1 is expressed not only in LVs but also in BVs and macrophages.[20] Contrary to wild-type (WT) mice, the mRNA levels of NOTCH1, Dll4, and Podoplanin of HIF-1α CKO mice were not up-regulated by DE stress (Fig 4A. NOTCH1: p = 0.231; Dll4: p = 0.127; Podoplanin: p = 0.246). Also, under desiccating stress, HIF-1α CKO mice showed significantly lower expression of NOTCH1 and Dll4 as compared to WT DE mice (Fig 4A, NOTCH1: p = 0.007; Dll4: p = 0.018). Although LYVE-1 expression of HIF-1α CKO mice increased after DE induction (p = 0.006), the average mRNA fold change was significantly lower as compared to that of DE-induced WT mice (p = 0.0497). Likewise, immunoblot and densitometry detected a downregulation of NOTCH1 and LYVE-1 in DE-induced HIF-1α CKO mice (Fig 4B). The reduced LYVE-1^+^ cell expression was confirmed by immunofluorescence staining in DE-induced HIF-1α CKO mice (Fig 4C).

### CD45^+^ cell infiltration increases after inhibition of lymphangiogenesis in LGs in DE

To study the role played by lymphangiogenesis in immune cell infiltration in LGs, the change of CD45^+^ cells in LGs during DE induction was measured using IHC staining and flow cytometry. Among inflammatory cell markers, CD45 antigen (leukocyte common antigen) was chosen because it is universally expressed in almost all hematolymphoid cells, including T lymphocytes, B lymphocytes, granulocytes, monocytes, and macrophages.[21] Therefore, the analysis of CD45^+^ cell infiltration in LGs may provide an understanding of the overall change of inflammatory status of DE induced LGs.

**Fig 5:**
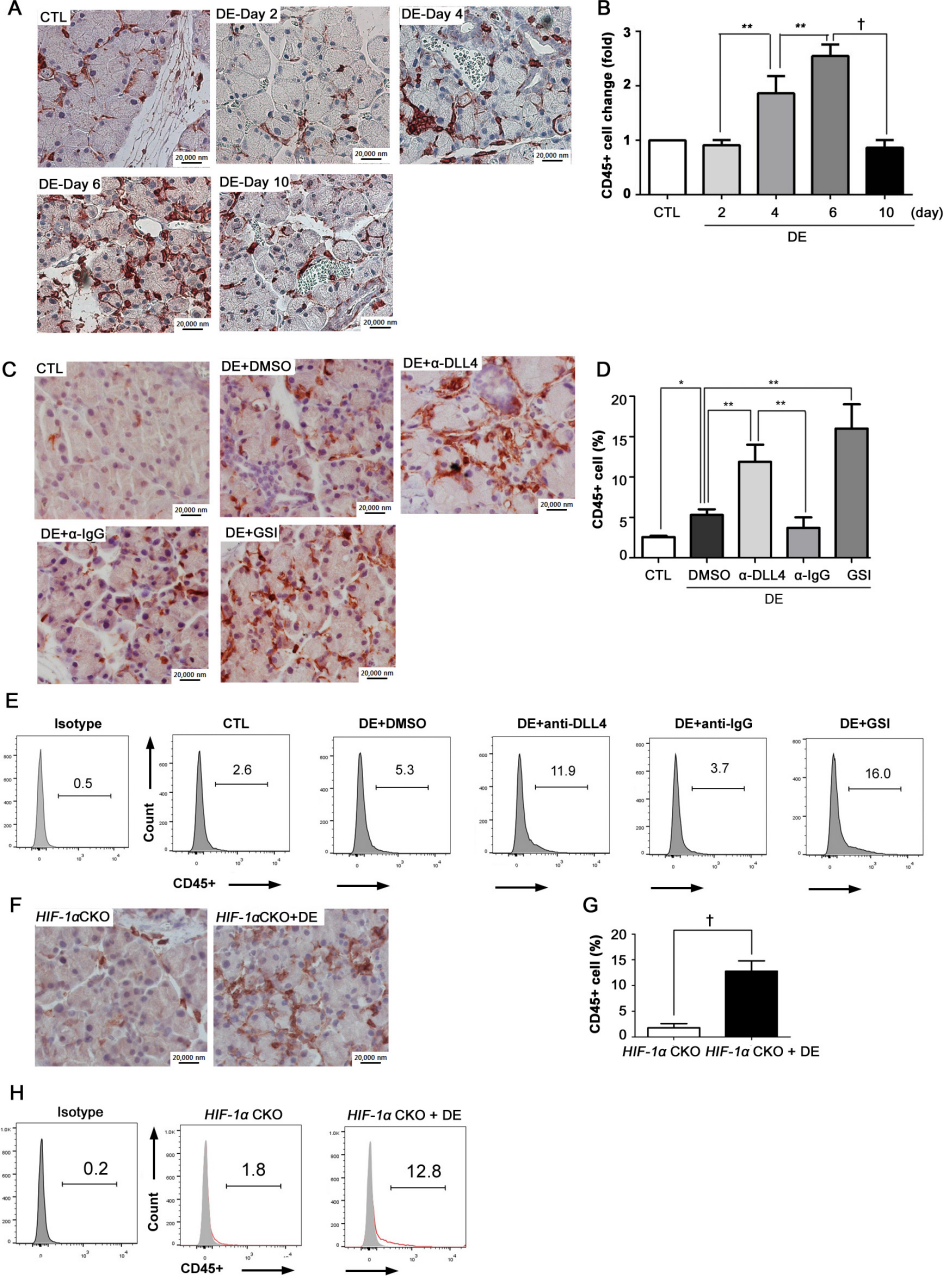
Change in CD45^+^ cells in dry eye-induced lacrimal glands. During 10 days of DE induction, (A) LGs were obtained on Days 0, 2, 4, 6, and 10 for IHC staining of CD45^+^ cells. (B) The fold change of CD45^+^ cells was measured at Days 0, 2, 4, 6, and 10. (C) During DE induction, several groups of mice were administered anti-Dll4 Ab and GSI to inhibit the NOTCH1-DLL4 axis. Anti-IgG Ab was administered as a control for the anti-Dll4 Ab group, and DMSO was administered as a negative control. LGs were obtained at Day 10 of DE induction and were prepared for IHC staining of CD45^+^ cells. (D) The actual percentage of CD45^+^ cells was calculated. (E) Flow cytometry for CD45^+^ cells was performed for each condition according to the manufacturer’s protocol. (F) DE was induced for 10 days in B6 and HIF-1CKOmice. LGs were obtained and prepared for IHC staining. (G) The actual percentage of CD45^+^ cells was calculated for non-DE HIF-1α CKO mice and the DE HIF-1α CKO mice. (H) Flow cytometry was performed for the two groups. Student’s t-test for statistical analysis: *p<0.05, **p<0.01, †p<0.001. Error bars indicate standard deviation. (CTL = normal control; DE = dry eye; α-Dll4 = anti-Dll4 antibody; α-IgG = anti-IgG antibody; GSI = γ-secretase inhibitor; DMSO = dissolved dimethyl sulfoxide; HIF-1α CKO = HIF-1α conditional knockout).

During DE induction, CD45^+^ cells increased, reaching the highest peak at Day 6. At Day 10, CD45^+^ cells decreased drastically (p = 0.00085), reaching almost the same level as the control group (Fig 5A & 5B). According to the flow cytometry data, the actual percentage of CD45^+^ cells changed from 3.5% (Day 0) to 3.8% (Day 10) when DE was induced in WT mice (data not shown).

Next, CD45^+^ cells were measured for DE-induced LGs after inhibiting Dll4/Notch. CD45^+^ cells were much infiltrated in DE-induced LGs with anti-Dll4 antibody or GSI injection as compared to the DMSO group (Fig 5C–5E, anti-Dll4 antibody: p = 0.0056; GSI: p = 0.0039). The actual percentage of CD45^+^ cell population was significantly greater than the DMSO group in both anti-Dll4 antibody and GSI group (Fig 5E, anti-Dll4 antibody: 11.9%, p = 0.0067; GSI: 16.0%, p = 0.0038). In IHC staining, CD45^+^ cells were diffusely scattered in the interlobular spaces and periductal areas (Fig 5A & 5C).

Lastly, CD45^+^ cell increased significantly in DE-induced HIF-1α CKO mice according to IHC staining (Fig 5F & 5G). Also, the actual percentage of CD45^+^ cells was 12.8% for DE-induced HIF-1α CKO mice, which was significantly higher than 1.8% of HIF-1α CKO without DE induction (Fig 5H).

## Discussion

**Fig 6:**
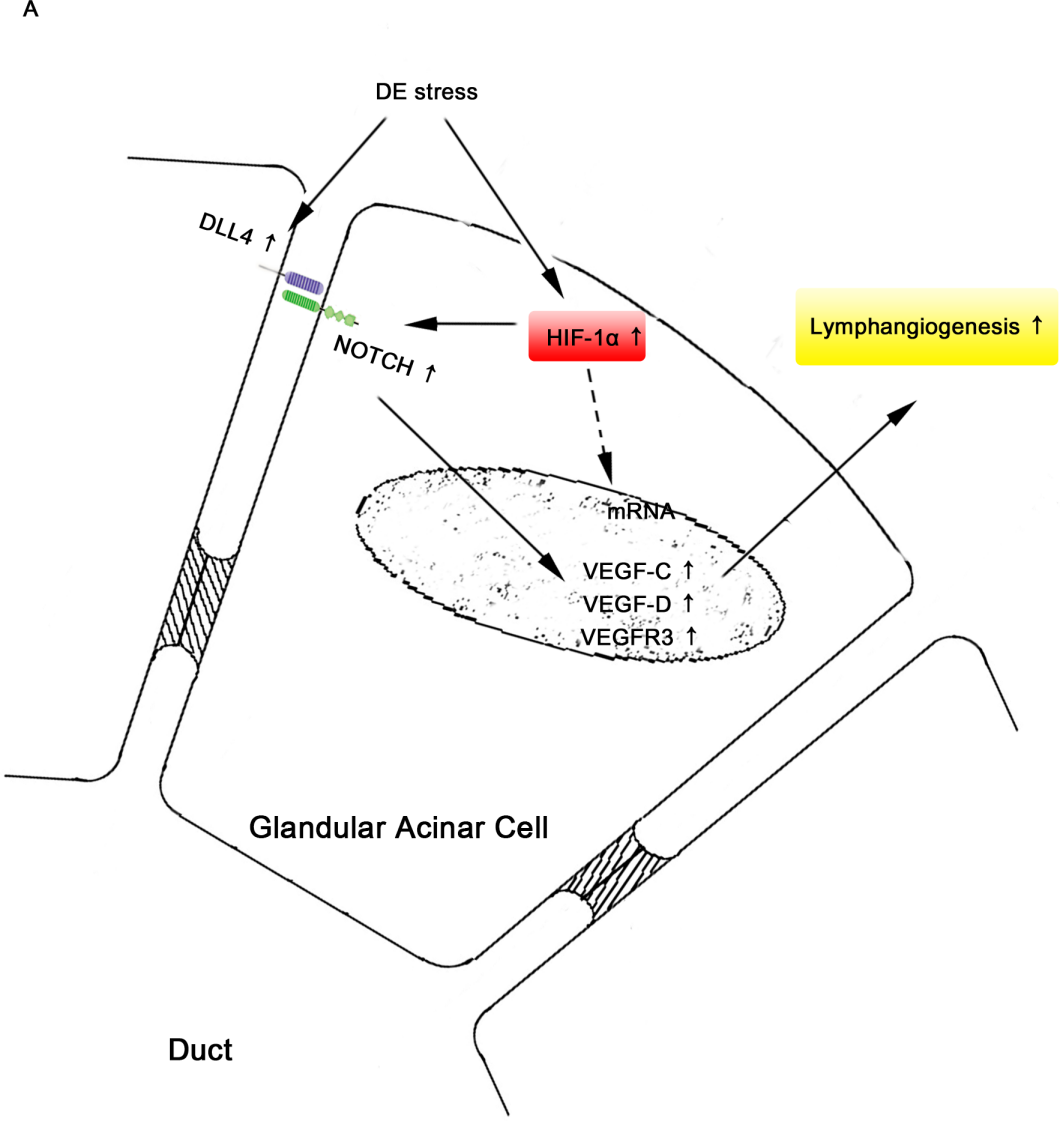
Dry eye stress activates NOTCH1-Dll4 axis and HIF-1α during lymphatic vessel formation of dry eye-induced lacrimal glands. DE stress activates Dll4/Notch pathway and HIF-1α in LGs. HIF-1α upregulates Dll4/Notch pathway and promotes lymphangiogenesis. Activation of Dll4/Notch pathway and HIF-1α results in increase of VEGF-C, VEGF-D, and VEGFR3, which results in lymphangiogenesis in LGs. (DE = dry eye).

The novel findings of this study are as follows: (1) Expression levels of the Dll4/Notch signaling pathway and lymphangiogenesis are significantly upregulated in DE-induced LGs. (2) HIF-1α is important for Dll4/Notch induced lymphangiogenesis in LGs of DE. (3)Lastly, the suppression of lymphangiogenesis significantly increases CD45^+^ cell infiltration in DE-induced LGs. A schematic figure summarizing the results of this study is shown in Fig 6.

### Effects of Dll4/Notch signaling on lymphangiogenesis in LGs in DE

The results of this study proved Dll4/Notch-induced lymphangiogenesis in DE LGs by qPCR, immunoblot and immunofluorescence staining (Figs 1–3). Similar to our results, Notch induced lymphangiogenesis has been reported by several previous works. Fatima et al. demonstrated the downregulation of VEGF-C and VEGFR3 in Notch-1 mutant lymphatic endothelial cells.[22] In addition, Niessen et al. manifested the downregulation of VEGF-C/VEGFR3 signaling by blocking Dll4/Notch 1 pathway in mouse dermis model.[17] Therefore, these previous results support the lymphangiogenic role of Dll4/Notch signaling as it has been demonstrated in this study.

Meanwhile, we could not determine which Notch receptors and ligands are exactly responsible for lymphangiogenesis from the present work. However, among the members of the Notch family, the mRNA levels of NOTCH1, NOTCH2, Dll4, Jagged1, and Jagged 2 were increased by DE stress. Yet, the mRNA fold increase of NOTCH1 and Dll4 was significantly higher than other Notch receptors and ligands (Fig 1A). Immunoblot assay also revealed high expression of NOTCH1 and Dll4 after DE stress (Fig 1C). Therefore, Dll4/Notch 1 may be the main Notch subtype in inducing lymphangiogenic pathways. Moreover, earlier studies have pointed to Dll4/Notch 1 signaling in particular as the main subtype of Notch signaling related to lymphangiogenesis.[17, 22, 23]

As opposed to the findings of previous studies where Dll4/Notch 1 signaling mainly regulated VEGF-C/VEGFR3 expression, the results of this study show that VEGF-C is subtly affected by the inhibition of Notch signaling by GSI or anti-Dll4 antibody. Per contra, VEGF-D was inhibited with good significance (Fig 3A). These results imply that Dll4/Notch 1 regulates VEGF-D/VEGFR3 expression rather than VEGF-C/VEGFR3 expression in the process of DE-induced lymphangiogenesis.

Another interesting finding is that the mRNA levels of LYVE-1 showed biphasic peak, at Day 4 and Day 10 of DE induction (Fig 2A). However, by the treatment of GSI or anti-Dll4 antibody, LYVE-1 expression was inhibited only at Day 10 (Fig 3). Although we could not unveil which cells were responsible for the early LYVE-1 expression, it might be caused by the LYVE-1^+^ bone marrow derived cells rather than by the lymphatic endothelium. Previous studies showed increased LYVE-1 expression by activated macrophages of various tissues in response to inflammation.[20, 24] Our data also shows early CD45^+^ cell infiltration in DE induced LGs (Fig 5A & 5B), which may demonstrate potentials for LYVE-1^+^ cell infiltration at Day 4. Additionally, despite the fact that mRNA level exhibited a high peak at Day 4, the actual LVs were found at Day 10 with immunostaining, but not at Day 4 (Fig 2C). This suggests that four days induction may be insufficient for forming mature LVs, implying that the mRNA peak at Day 4 does not represent LV structure. Future studies for identifying the exact identity of the early mRNA expression of LYVE-1 will be performed for clarification of this finding.

### HIF-1α regulates Dll4/Notch signaling and lymphangiogenesis in DE-induced LGs

In DE induced HIF-1α CKO mice, the expressions of DLL4, NOTCH1, LYVE-1, and Podoplanin were inhibited (Fig 4). This indicates that Dll4/Notch signaling and LV formation is regulated by HIF-1α. Bridges et al. supported HIF-1α regulated Notch signaling in lung epithelium, where Notch signaling and lymphangiogenesis was increased by up-regulation of HIF-1α.[13] In addition, VEGFs, transcribed by the activation of HIF-1α, have been shown to interact with Notch activated VEGFR3 to form new LVs.[25, 26] Indeed, our data shows that HIF-1α knockout suppresses Notch signaling and lymphangiogenesis. However, since we did not investigate intracellular signaling for Dll4/Notch induction by HIF-1α, the temporal relationship between HIF-1α and Notch signaling in LGs requires further evaluation.

### LVs help reduce inflammatory cell infiltration in DE-induced LGs

The results of this study show diminished CD45^+^ cell infiltration after the completion of LV formation, while blocking lymphangiogenesis enhanced CD45^+^ cell infiltration. These findings indicate that newly formed LVs help reduce inflammatory cells in DE induced LGs.

In previous studies, lymphangiogenesis has been helpful in resolving inflammation induced tissue damage in inflammatory diseases.[27, 28] In skin inflammatory disease, lymphangiogenesis regulated fluid drainage, immune cell migration, and the removal of inflammatory cells, thereby accelerating the resolution of inflammation.[29, 30] Additionally, VEGF-C/VEGFR3 induced lymphangiogenesis accelerated clearance of inflammatory cells and bacterial antigens from inflamed colon to draining lymph nodes in inflammatory bowel disease.[31] Likewise, the results of this study show that lymphangiogenesis during DE-induction aid the resolution of DE-induced inflammation by clearing CD45^+^ cells from LGs.

There are some limitations of this study. The mouse model for investigating DE may be quite different from the human DE. Human pathologic studies will be needed to confirm the role of Notch signaling induced lymphangiogenesis of LGs. Also, scopolamine, which was used as a routine protocol for DE induction, may have altered the expression of Dll4/Notch pathway and may have affected our results. In addition, while we have only focused on HIF-1α and Notch signaling, other known pathways related to lymphangiogenesis, such as nuclear factor-kappaB (NF-κB) and Janus kinase/signal transducers and activators of transcription (JAK/STAT), may have been activated by DE stress and may have influenced our results.[26, 32] Future studies with these major pathways are needed to fully understand LV formation in DE LGs.

In conclusion, this study demonstrates the relationship between Dll4/Notch signaling, HIF-1α activation, and lymphangiogenesis in LGs in DE. By aiding the reduction of inflammatory cells from LGs, LVs are important in lessening inflammatory damage. Further study for investigating the neural network between the cornea and the LG may explain more fully how the Notch system is activated in LGs. Moreover, cross-talk between VEGFs, Notch signaling, and HIF-1α should be investigated in order to clarify lymphangiogenesis in DE-induced LG.

## Supporting Information

S1 Fig Primer sequences of the preformulated primers used in qRT-PCR. (Figure A) Primer sequences of the preformulated primers used in qRT-PCR are listed as follows.
(TIF)

S2 Fig Change in LYVE-1 expression at Day 4 of dry-eye induction after inhibition of the NOTCH1-Dll4 axis by intraperitoneal injection of monoclonal anti-Dll4 antibody and γ-secretase inhibitor. While C57BL/6 mice were housed in a CEC with scopolamine administration, several groups of mice were administered anti-Dll4 Ab and GSI for inhibition of the NOTCH1-DLL4 axis. Anti-IgG Ab was administered as a control for the anti-Dll4 Ab group, and DMSO was administered as a negative control. LGs were obtained after 4 days of DE induction. (Figure A) The mRNA level of LYVE-1 was measured using qPCR. Student’s t-test for statistical analysis: *p<0.05. Error bars indicate standard deviation. (CTL = normal control; DE = dry eye; α-Dll4 = anti-Dll4 antibody; α-IgG = anti-IgG antibody; GSI = γ-secretase inhibitor; DMSO = dissolved dimethyl sulfoxide).
(TIF)
