# Supplementary figures and images for: Activation of Dll4/Notch Signaling and Hypoxia-Inducible Factor-1 Alpha Facilitates Lymphangiogenesis in Lacrimal Glands in Dry Eye
Source: PLoS One. 2016 Feb 1;11(2):e0147846. doi: 10.1371/journal.pone.0147846 (PMC4734677; doi:10.1371/journal.pone.0147846)

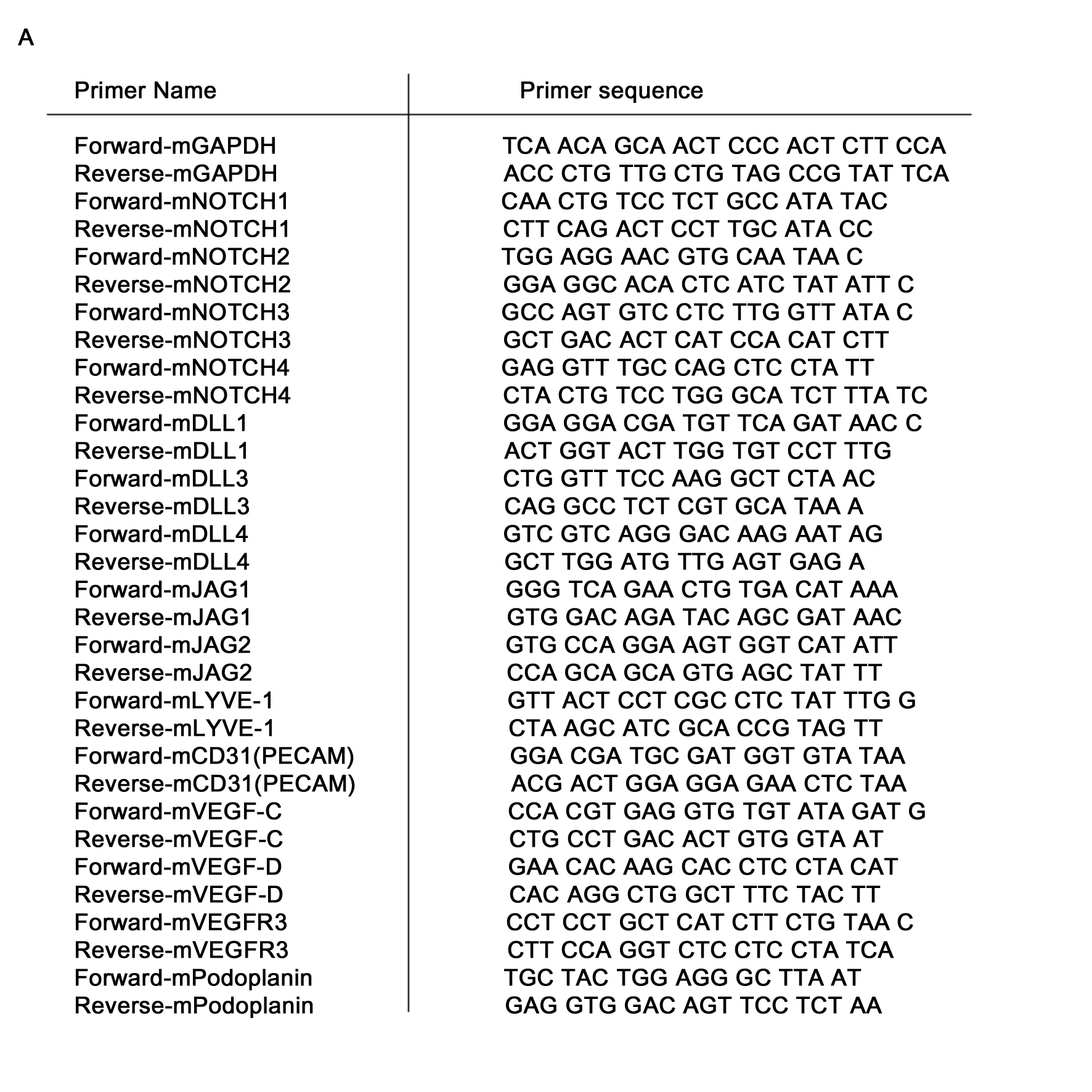

Supplement: S1 Fig — (Figure A) Primer sequences of the preformulated primers used in qRT-PCR are listed as follows. (TIF) [file pone.0147846.s001.tif]

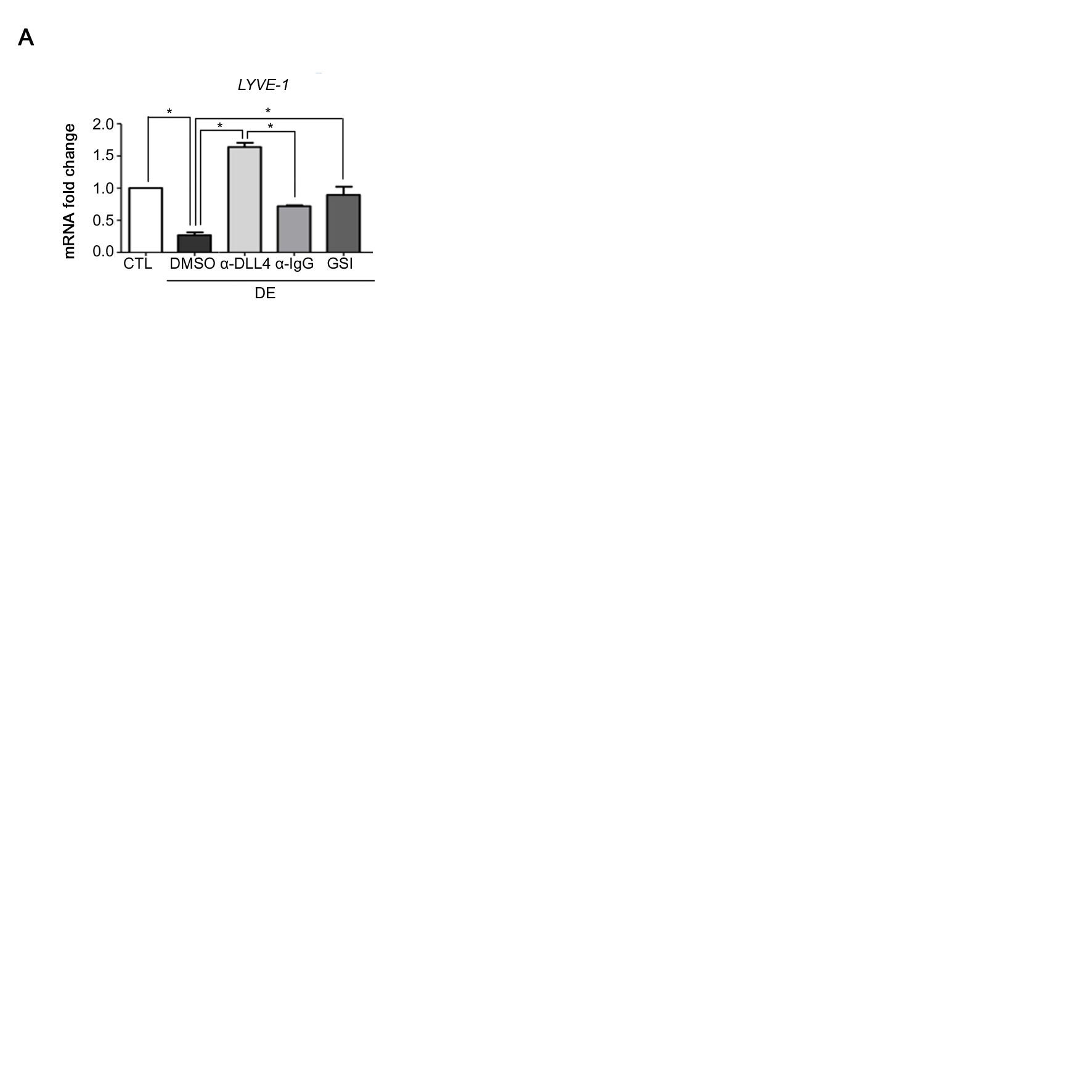

Supplement: S2 Fig — While C57BL/6 mice were housed in a CEC with scopolamine administration, several groups of mice were administered anti-Dll4 Ab and GSI for inhibition of the NOTCH1-DLL4 axis. Anti-IgG Ab was administered as a control for the anti-Dll4 Ab group, and DMSO was administered as a negative control. LGs were obtained after 4 days of DE induction. (Figure A) The mRNA level of LYVE-1 was measured using qPCR. Student’s t-test for statistical analysis: *p<0.05. Error bars indicate standard deviation. (CTL = normal control; DE = dry eye; α-Dll4 = anti-Dll4 antibody; α-IgG = anti-IgG antibody; GSI = γ-secretase inhibitor; DMSO = dissolved dimethyl sulfoxide). (TIF) [file pone.0147846.s002.tif]
